# Screening of *Streptococcus suis* in swine workers of selected states in Peninsular Malaysia

**DOI:** 10.14202/vetworld.2024.1-7

**Published:** 2024-01-04

**Authors:** Chee Yien Lee, Zunita Zakaria, Gayathri Thevi Selvarajah, Farina Mustaffa-Kamal, Kenny Gah Leong Voon, Michelle Wai Cheng Fong, Peck Toung Ooi

**Affiliations:** 1 Department of Veterinary Clinical Studies, Faculty of Veterinary Medicine, Universiti Putra Malaysia, 43400 UPM Serdang, Selangor, Malaysia; 2 Department of Veterinary Pathology and Microbiology, Faculty of Veterinary Medicine, Universiti Putra Malaysia, 43400 UPM Serdang, Selangor, Malaysia; 3 Laboratory of Vaccine and Biomolecules, Institute of Bioscience, Universiti Putra Malaysia, 43400 UPM Serdang, Selangor, Malaysia; 4 UPM - MAKNA Cancer Research Laboratory, Institute of Bioscience, Universiti Putra Malaysia, 43400 UPM Serdang, Selangor, Malaysia; 5 Division of Biomedical Science, School of Pharmacy, University of Nottingham, 43500 Semenyih, Selangor, Malaysia

**Keywords:** carrier, high-risk occupation, human, *Streptococcus suis*

## Abstract

**Background and Aim:**

*Streptococcus suis* is a zoonotic pathogen that is highly associated with contact between live pigs and raw pig material. In view of the recent reports of human infections in Malaysia, epidemiological data on the status of *S. suis* in the human population, especially among people working closely with pigs and/or raw pork, should be provided. The aim of this study was to detect *S. suis* among individuals working in the swine industry in several major pig production areas in Peninsular Malaysia.

**Materials and Methods:**

Demographic information, exposure determinants, and oral swabs were collected from swine personnel, including farmers, butchers, and veterinarians. Oral swabs were subjected to bacterial isolation and conventional polymerase chain reaction (PCR) assays for *S. suis* detection.

**Results:**

The study included 40 participants working in the swine industry, with a predominant representation of males (62.5%) and Malaysian Chinese individuals (60.0%) who consumed pork (92.5%). Notably, none of the participants reported consuming raw or partially cooked pork. In spite of their occupational exposure risk, none of the oral swabs showed positive results for *S. suis* infection.

**Conclusion:**

To the best of our knowledge, this is the first report and detection study of *S. suis* using oral swabs obtained from swine personnel in Peninsular Malaysia.

## Introduction

*Streptococcus suis* are facultative anaerobic Gram-positive cocci bacteria encapsulated by a polysaccharide layer responsible for evading the host cell immune system [1]. Capsular polysaccharide composition and structure are well-known characteristics of *S. suis* that distinguish them into serotypes [2]. It is a major porcine pathogen that causes fatal and economic losses worldwide in the swine industry. *S. suis* is also an important zoonotic pathogen that causes severe systemic disease in people who are in contact with infected pigs or contaminated pork products [3]. Human infections commonly present with meningitis or septicemia, and other reported infections include endocarditis, arthritis, cellulitis, spondylodiscitis, rhabdomyolysis, pneumonia, peritonitis, uveitis, and endophthalmitis [4–6]. Over the last two decades, the number of reported cases of *S. suis* in humans has increased significantly. Some of these cases are related to occupations such as veterinarians, pig farmers, pig breeders, abattoir workers, meat inspectors, and butchers [7, 8]. Transmission of *S. suis* from pigs to humans can occur through close contact, cuts or abrasions on skin, pig bite, raw or undercooked pork consumption, or even pig blood [3, 9, 10]. *S. suis* aerosolization in a swine-confined building also poses occupational exposure to farm workers [11]. Outbreaks of *S. suis* infection can be sporadic or stem from a single source of infection [12, 13]. Some cases have also been reported without prior contact with live pigs or related products [14–16].

Although the pork is consumed by only a minority of the population due to religious beliefs, it contributes significantly to the Malaysian livestock sector. According to Malaysian statistics, pork is consumed by approximately 36.5% of the population and the per capita consumption is estimated to be 17.3 kg based on a 12 million non-Muslim population for 2021 [17]. Two *S. suis* infections in humans have recently been reported in Sabah, Malaysia, involving two men with a history of contact with pigs. One had daily contact with pigs, while the other was a butcher who had an injured thumb 2 days before the onset of symptoms [18]. This report has led to the need to carry out further screening of *S. suis* among persons working closely with pigs.

*S. suis* colonizes the upper respiratory tract, gastrointestinal tract, and genital organs of a wide variety of animals, including pigs, cats, dogs, ruminants, deer, and horses [19]. *S. suis* is well known to be a normal flora in the tonsils of animals, particularly pigs [20]. Capsular polysaccharide expression, adhesin formation, and biofilm formation are the few demonstrated mechanisms supporting its survival on the host mucosal surface [21–25]. *Streptococcus* species, such as *Streptococcus mitis* and *Streptococcus salivarius*, are known to inhabit the oral cavity of humans as early as birth [26]. Similar to *S. suis* in pigs, oral streptococci colonize the oral cavity with high-affinity adhesins and form a complex community with another commensal [26]. Studies investigating *S. suis* carrier status among abattoir employees, swine producers, and meat processing workers have revealed very low detection rates from the tonsillar, nasal, and pharyngeal samples [11, 27, 28]. However, there is still a gap in the carrier status of *S. suis* in healthy humans. In addition, biochemical differentiation of *S. suis* from other streptococci presents great challenges. The prevalence of *S. suis* was thought to be underestimated because *S. suis* is commonly misidentified as other *Streptococcus* species such as *Streptococcus* spp., *Streptococcus bovis*, *Streptococcus agalactiae*, *Streptococcus anginosus*, *Streptococcus sanguinis*, *Streptococcus viridans* and *S. mitis* groups [25, 29, 30].

Because information on whether *S. suis* may be harbored in the oral cavity of swine workers in Malaysia is still obscure, this study aimed to detect *S. suis* in the oral cavity of humans, especially those working in swine-related fields in Peninsular Malaysia. To further identify the risk of *S. suis* infection, exposure determinants, such as duration of work, pork consumption, and hand injury, were explored.

## Materials and Methods

### Ethical approval and Informed consent

The Ethics Committee for Research Involving Human Subjects, Universiti Putra Malaysia (JKEUPM-2020-104) approved this study. All participants provided consent to participate in the study, and their identities were kept confidential.

### Study period and location

This study was conducted from November 2021 to April 2022. Sampling locations from the West Coast of Peninsular Malaysia were selected on the basis of the area being a main pig farming area with confirmed cases of swine *S. suis* and with access permission granted by the participants.

### Sampling

In this study, convenience sampling and purposive sampling methods were employed. Participants were selected on the basis of the inclusion and exclusion criteria listed in Table-1.

**Table-1:** Inclusion and exclusion criteria.

Inclusion criteria	Exclusion criteria
• Adult >18 years old.	• Received antibiotic treatment for the past 14 days.
• Working with live pigs or raw pork materials at a minimum 8 contact hours a week (e.g., swine farm workers, pork butchers, swine veterinarians, etc.).	• Positive for COVID-19. • Refusal to give informed consent.

Each participant was interviewed to obtain information on their demographics (age, ethnicity, nationality, and occupation) and exposure determinants (duration of work in the swine industry, consumption of raw or undercooked pig products). Other basic data, such as gender and occupation, were also recorded. At the same time, wounds were observed on both hands of the participants. Oral swabs were collected from the participants using a flocked swab (Copan, USA) and kept chilled (4°C–8°C) in Amies transport media (Labchem, Malaysia) until further processing in the Clinical Laboratory of Faculty of Veterinary Medicine, Universiti Putra Malaysia.

### Bacteria isolation

Swabs were inoculated into Columbia sheep blood agar (Oxoid, USA) and *Streptococcus* selective agar (colistin-bacitracin supplemented agar-COBA) and incubated at 37 °C for 24–48 h, depending on the bacterial growth. Bacterial colonies exhibiting alpha hemolysis were selected for subculture to obtain pure colonies. Pure colonies were then stained with Gram staining and subjected to catalase test as a pre-screening test for *S. suis* to identify Gram-positive and catalase-negative colonies.

### DNA extraction and polymerase chain reaction (PCR) amplification

Pure colonies were suspended in 100 μL of autoclaved deionized water. Oral swabs were simultaneously suspended in 100 μL of autoclaved deionized water. DNA was extracted from bacterial and swab suspensions using a column-based commercial DNA extraction kit (DNeasy Blood and Tissue Kit, Qiagen, Germany) according to the manufacturer’s protocol. The extracted DNA was subjected to *S. suis* detection using two sets of conventional polymerase PCR assays with species-specific primers previously published by Kerdsin *et al*. [31] and Ishida *et al*. [32], which detected the *gdh* and *recN* genes, respectively. The polymerase chain reaction (PCR) assay reaction setup consisted of 12.5 μL of Redmix PCR master mix (Meridien Bioscience, Korea), 1 μL of 10 mM primer each, and 2 μL (50–100 ng) of extracted DNA. Cycling conditions were optimized according to the PCR reagents and set at 94°C for 3 min, 30 cycles of 94°C for 30 s, 57 for 1 min, and 72 for 1 min, and 72°C for 10 min. Polymerase chain reaction products were electrophoresed for 40 min at 80 V using pre-stained (Redsafe™ nucleic acid staining solution, iNtRON Biotechnology, Korea), 2% agarose gel in tris-acetate ethylenediaminetetraacetic acid buffer. An ultraviolet transilluminator was used to view the electrophoresed gel. Samples were considered positive if the *gdh* (695 bp) and *recN* (325 bp) genes of *S. suis* were detected.

### Statistical analysis

Statistical analyses were performed using the statistical software Minitab version 21 (https://www.minitab.com/en-us/products/minitab/) and only descriptive statistical analyses were performed. Continuous data (i.e., age and duration of work) are presented as mean ± standard deviation (SD) and median, whereas categorical data (i.e., gender, occupation, pork consumption, and hand injury) are presented as frequencies and percentages.

## Results

**Figure-1:**
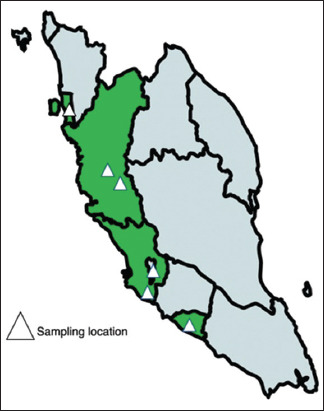
Mapping location of samples collected. Exact locations of sampling conducted were pinned by a triangle icon [Source: Map was created using an online tool available at https://www.mapchart.net/index.html].

A total of 40 swine industry personnel working as farm workers (n = 30), veterinarians (n = 8), and butchers (n = 2) from the states of Penang (n = 2), Perak (n = 12), Selangor (n = 22), and Malacca (n = 4) were enrolled in the study, as shown in Figure 1. Among the participants, 60.0% (24/40) were local Malaysians of Chinese ethnicity and 40.0% (16/40) were foreigners residing and working in Malaysia. A total of 25 male and 15 female participants aged 22–60 years (mean and median age, 32.6 and 30 years, respectively). The mean working experience in the swine industry was 7.42 (SD, 6.67) years, with the longest working duration being 25 years.

**Figure-2:**
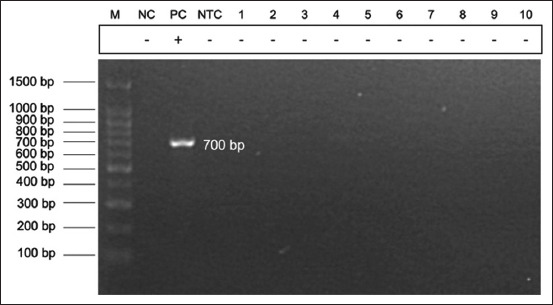
Electrophoresed gel photo of *Streptococcus suis* *gdh* gene (expected 695 bp) polymerase chain reaction assay. M: 100 bp DNA ladder, NC: Negative control (*Streptococcus dysgalactiae*), PC: positive control (*S. suis* of swine field isolate), NTC: No template control, Lanes 1–10: samples H1–H10 (all negative).

**Figure-3:**
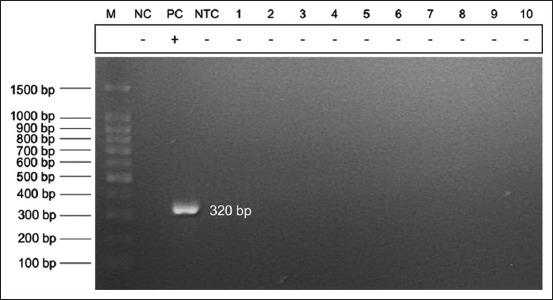
Electrophoresed gel photo of *Streptococcus suis* *recN* gene (expected 325 bp) polymerase chain reaction assay. M: 100 bp DNA ladder, NC: negative control (*Streptococcus dysgalactiae*), PC: Positive control (*S. suis* of swine field isolate), NTC: No template control, Lanes 1–10: samples H1–H10 (all negative).

Most of the participants ate pork (92.5%), but none of them consumed raw or partially cooked pork. All of the participants did not display any clinical syndrome indicating *S. suis* infection, such as malaise, high fever, nausea, and vomiting, and only 15% of the participants had skin injuries on their hands on sampling. Supplementary Table-1 lists all demographic information and exposure determinants of each participant. Forty oral swab samples were collected. Alpha-hemolytic streptococcus-like bacterial colonies were present on both COBA and sheep blood agar after 24 h of incubation, but all bacterial samples were negative for both *gdh* and *recN* genes of *S. suis* (Figures-2 and 3). Similarly, PCR assay of DNA extracted from oral swab suspensions yielded negative results for all samples.

**Supplementary Table-1:** Demographic information and exposure determinants of each participant.

ID	Age (year)	Ethnic/Nationality	Gender	Location (state)	Occupation	Estimated working duration (year)	Eat pork	Wound on hand
H01	56	Chinese	F	Perak	Butcher	19	Yes	Yes
H02	59	Chinese	M	Perak	Butcher	25	Yes	Yes
H03	44	Chinese	M	Selangor	Veterinarian	20	Yes	No
H04	29	Chinese	F	Selangor	Veterinarian	5	Yes	No
H05	29	Chinese	F	Selangor	Veterinarian	3	Yes	No
H06	34	Chinese	F	Selangor	Veterinarian	10	Yes	No
H07	44	Chinese	M	Malacca	Farm worker	20	Yes	No
H08	22	Foreigner	M	Malacca	Farm worker	3	Yes	No
H09	25	Foreigner	M	Malacca	Farm worker	3	Yes	No
H10	24	Foreigner	M	Malacca	Farm worker	3	Yes	No
H11	26	Chinese	M	Penang	Veterinarian	4	Yes	No
H12	25	Chinese	M	Selangor	Veterinarian	4	Yes	No
H13	30	Chinese	M	Penang	Veterinarian	2	Yes	No
H14	29	Chinese	M	Selangor	Veterinarian	6	Yes	Yes
H15	31	Chinese	M	Selangor	Farm worker	6	Yes	No
H16	35	Chinese	M	Selangor	Farm worker	6	Yes	No
H17	25	Chinese	M	Selangor	Farm worker	10	Yes	No
H18	25	Chinese	M	Selangor	Farm worker	10	Yes	No
H19	27	Chinese	M	Selangor	Farm worker	6	Yes	No
H20	30	Chinese	M	Selangor	Farm worker	10	Yes	No
H21	20	Chinese	F	Selangor	Farm worker	2	Yes	No
H22	45	Chinese	F	Selangor	Farm worker	6	Yes	No
H23	38	Foreigner	M	Selangor	Farm worker	5	Yes	No
H24	60	Chinese	M	Selangor	Farm worker	23	Yes	No
H25	23	Foreigner	M	Selangor	Farm worker	5	Yes	No
H26	34	Foreigner	F	Selangor	Farm worker	8	Yes	No
H27	44	Foreigner	M	Selangor	Farm worker	8	Yes	No
H28	35	Foreigner	M	Selangor	Farm worker	3	Yes	No
H29	31	Foreigner	M	Selangor	Farm worker	3	Yes	No
H30	32	Foreigner	F	Selangor	Farm worker	5	Yes	No
H31	31	Foreigner	M	Perak	Farm worker	12.5	Yes	No
H32	25	Foreigner	M	Perak	Farm worker	8	No	No
H33	42	Foreigner	M	Perak	Farm worker	7	Yes	No
H34	44	Foreigner	F	Perak	Farm worker	6	Yes	No
H35	26	Foreigner	F	Perak	Farm worker	7	No	Yes
H36	30	Foreigner	F	Perak	Farm worker	7	No	No
H37	25	Chinese	F	Perak	Farm worker	<1	Yes	Yes
H38	25	Chinese	F	Perak	Farm worker	<1	Yes	Yes
H39	23	Chinese	F	Perak	Farm worker	<1	Yes	No
H40	23	Chinese	F	Perak	Farm worker	<1	Yes	No

F = Female, M = Male

## Discussion

Based on the demographic data collected, pig farming in Peninsular Malaysia relied greatly on foreign workers (16/30 farm workers) as the agricultural work is considered “3D” (dirty, dangerous, and difficult) with low pay and limited job prospects, hindering local people from participating in this agriculture industry [33, 34]. In addition to foreign workers, the Malaysian Chinese ethnic group (24/40) remained dominant in the swine industry, including non-professionals such as farm owners or farm workers, and professionals such as veterinarians. Because pig farming in Malaysia was started by Chinese ethnic groups, it has been maintained as a family business [35]. Chinese ethnic group is also the main consumer of pork in Malaysia [36].

Cases of *S. suis* infections have been observed notably in the United States, the United Kingdom, China, Vietnam, and Thailand [37], where pig farming and high pork consumption are major sources of pork production. Disparities can be seen between Western countries and some Asian countries in terms of lifestyle, the widespread utilization of backyard production systems, and the close interaction of diverse animal species, including humans, with pigs, which contribute to a higher number of cases in Asia [38]. Most cases in Western countries are associated with occupational contact with pigs or pork products [18, 37]. The incidence of *S. suis* was also significantly higher in the occupational group than in the general population [39]. The previous reports by Feng *et al*. [9] and Goyette-Desjardins *et al*. [10] indicated that swine-related workers with open wounds (such as cuts and abrasions) were also more susceptible to *S. suis* infections and in this study, there were only a few of the participants (15%) had injuries on their hand. Interestingly, this study was conducted during the COVID-19 pandemic; everyone wore a face mask and practiced constant hand sanitation. The high awareness of personal hygiene might have reduced the risk of infection through the cutaneous route.

In some Asian countries, consumption of raw and undercooked pork, fermented raw pork, blood, and offal products in customary local dishes is the main cause of human infection [40]. However, as in our study, pork consumers in Malaysia generally do not consume raw or partially cooked pork. This significantly reduces the risk of oral route infection, which explains why no *S. suis* was detected among the participants. Furthermore, when participants were asymptomatic, it was very unlikely to detect *S. suis*, as previously documented [41], indicating that oral swabs may not be a good surveillance tool for the detection of *S. suis*. However, this study did not collect serum samples, we could not determine whether actual infection or previous exposure occurred. However, when the oral swabs were negative for *S. suis*, alpha-hemolytic *Streptococcus*-like colonies were observed in the bacterial cultures on the *Streptococcus* selective agar. This finding was expected because *Streptococcus* species have been reported to be a normal flora of the human oral cavity [26] and molecular detection methods such as polymerase chain reaction (PCR) assays provide a quicker means for identification.

While conducting this research, there were great challenges, leading to fewer participants. Because the study was conducted during the COVID-19 pandemic, many swine personnel were concerned about contracting the disease and were not willing to participate. This was also a contributing factor because Selangor had the highest number of participants because movement restrictions were implemented during the study period, limiting access to other states. In addition, the Malaysian swine farming sector faced a negative threat from the outbreak of African swine fever (ASF) at that time, which further hampered the availability and acceptance of farms for research purposes.

Although *S. suis* was not detected in this study, few good practices were identified by swine personnel. The emergence of the COVID-19 pandemic and the outbreak of ASF increased personal hygiene and farm biosecurity measures, such as frequent washing of hands or showering before and after entry into farms. In addition, there were no participants from local and foreign workers eating uncooked pork products. To prevent *S. suis* infection in at-risk workers, workers should be educated about the importance of wearing proper protective gear when working with pigs, proper hand sanitation after handling pigs or pork, avoiding consumption of raw or undercooked pork, and seeking medical treatment as soon as possible if injury occurs during handling of pigs or pork.

## Conclusion

To the best of our knowledge, this is the first detection study of *S. suis* in swine personnel in selected states of Peninsular Malaysia. In addition, swine-related workers observed good hygiene and non-consumption of raw or undercooked pork which further reduced the risk of infection. Although no positive case was detected in the present study, *S. suis* remains a potential threat to related professions. Clinicians and microbiologists must be careful when diagnosing and interpreting microbiological results, in particular, if the patient has a history of contact with pigs or raw pork.

## Authors’ Contributions

CYL, PTO, ZZ, FM, GTS, and KGLV: Conceived and designed the study. CYL, PTO, and MWCF: Collected samples. CYL: Performed the experimental works. CYL and MWCF: Analysed and interpreted the data. CYL and PTO: Drafted and revised the manuscript. All authors have read, reviewed, and approved the final manuscript.
